# A Tribute to Philadelphians

**Published:** 1906-05

**Authors:** 


### A TRIBUTE TO PHILADELPHIANS.

Several southern dentists were the guests of the Philadelphia Dental Club at its thirty-first annual dinner. The following remarks of one of them, Dr. J. A. Chapple of Atlanta, Ga., which we were fortunate to secure shows that it must have been a grand affair, and that the speaker had a royal good time.—EDITOR.

"In the midst of such an atmosphere and this splendid presence the first thought that presents itself to the mind of the speaker is the fact that while modern dentistry had its birth in the Monumental city, and for long years stood as a mecca of the profession, the shrine has shifted just a little eastward and is to day enthroned in this City of Brotherly Love.

This slight geographical change is not due to any maudlin or false sentiment, but has been wrought strictly through and by the superb genius of authorship.

No other city in the world has furnished as many authors of dental literature as Philadelphia, while the varied multiplicity of mechanical design, the product of her sons, has revolutionized many of the methods of practice.

Here, also, lives the man whose oral surgery was the accepted standard throughout all christendom, and whose methods in certain operative procedure have not been supplanted even in this marvelous age of progress in general surgery.

It was here, too, where the greatest controversy that ever appeared in dental literature had its origin, when the "Apostle of Plastics" gave utterance to the condemnation of gold as a universal preserver of all conditions of tooth structure.

He stirred the dental world as it had never been aroused before, and the like of which has never occurred since. As a controversialist, Foster Flagg was preeminently at the head of the list, and sustained himself with admirable poise, and when he laid down the pen and "wrapped the drapery of his couch about him he bequeathed us an aphorism which has crystalized itself into a tremendous truth and which is being daily verified in the observation of all conservative men.

Moreover who among the profession at large was a more prolific contributor of dental literature than the lamented Burchard?

Is it a simple coincidence, or were the efforts of these men an inspiration to the present generation among you?

Whatever the cause, the fact—a stupendous fact—remains, which no amount of logic can dissipate, and that is that this godly old town still continues to be the literary hub of the universe. Indeed, it must have been a Philadelphian's presence who inspired the poet-philosopher to exclaim: "There is a child among you taking notes and faith and he will print them."

If these literary pioneers, who have gone to their final reward, shed such brilliant lustre upon the pages of dental literature, what fitting adjective can be employed to adequately characterize the efforts of those who "move and have their being" in your very midst to-day?

With perhaps the exception of one, every branch of our science has its acknowledged authority and champion in Philadelphia, while the exception noted is now in process of compilation and will be given to the profession at an early date.

When a great university confers the degree of LL. D. on a practitioner of dentistry, the laity pricked up its ears interrogatively, while the profession at large, at home and abroad, regard it "comme il faut," and heartly rejoiced with the object of the honor, an honor which should prove the crowning glory of a well spent life. And while this venerable patriarch may deplore his enforced retirement from an editorial work, and may never resume the chair again, he has long since achieved a glorious task and left the impress of his personality upon generations yet to come.

Were I to pause here, I should fail to make good the declaration that the dental shrine has been transferred hence, it needs only a cap-stone to make the ensemble complete, and we have it in the additional and incontrovertible fact that here, also, lives the greatest editor of the greatest dental journal in the world.

Aside from his specific duties as journalist, his achievements in literature, education and original research have easily placed him at the head of the entire dental host, and have wrung applause and recognition from even a foreign government. And yet they say "republics are ungrateful."

One of my graduated students, wishing to perfect himself in certain lines, appealed to me for advice.

"Go to Philadelphia," said I.

"But, Doctor, why not send me to Chicago?"

"Because they are a one-idead people in Chicago. Do you know, sir, that the dentists of this town have influenced the medical profession in prescribing porcelain pills as an hepatic stimulant? Go to Philadelphia, sir, the place for all doubting Thomases. After you will have registered, they will show you how to do things. I insist that you go to Kirk at least every Sunday. In due season your comprehension will be pearced by the hand of a Truman, and they will finally "jack you up' and 'put a head on you.'"

With this imperfect and kaleidoscopic review of the multiform of the accomplishments of our Philadelphia confreres, whose influence is seen, felt and heard in the remotest corners of the globe, it required no *second* invitation from the Philadelphia Dental Club to come up and "pay tribute to Caesar," swear allegiance to his cause and receive "absolution."

What you have achieved in the past or may accomplish in the future reflects itself, like the beneficent rays of the sun, upon all, and the profession at large becomes the beneficiary of your labor.

There are none who are jealous, none who would venture to pluck a laurel from your wreath or a penny from your exchecquer, but all rejoice with you in the precedence and excellence of your work, and invoke the divine approval of "Well done, good and faithful servants, in as much as ye have done it upon one of the least of these, ye have done it unto Me."

